# FPGA implemented testbed in 8-by-8 and 2-by-2 OFDM–MIMO channel estimation and design of baseband transceiver

**DOI:** 10.1186/s40064-016-1827-z

**Published:** 2016-03-31

**Authors:** S. Ramesh, R. Seshasayanan

**Affiliations:** Research scholar, Faculty of Electronics, Sathyabama University, Chennai, 600119 India; Department of ECE, Anna University, Chennai, 600025 India

**Keywords:** OFDM–MIMO, Channel estimation, IEEE 802.11a, Baseband transceiver, FPGA, Testbed

## Abstract

In this study, a baseband OFDM–MIMO framework with channel timing and estimation synchronization is composed and executed utilizing the FPGA innovation. The framework is prototyped in light of the IEEE 802.11a standard and the signals transmitted and received utilizing a data transmission of 20 MHz. With the assistance of the QPSK tweak, the framework can accomplish a throughput of 24 Mbps. Besides, the LS formula is executed and the estimation of a frequency-specific fading channel is illustrated. For the rough estimation of timing, MNC plan is examined and actualized. Above all else, the whole framework is demonstrated in MATLAB and a drifting point model is set up. At that point, the altered point model is made with the assistance of Simulink and Xilinx’s System Generator for DSP. In this way, the framework is incorporated and actualized inside of Xilinx’s ISE tools and focused to Xilinx Virtex 5 board. In addition, an equipment co-simulation is contrived to decrease the preparing time while figuring the BER of the fixed point model. The work concentrates on above all else venture for further examination of planning creative channel estimation strategies towards applications in the fourth era (4G) mobile correspondence frameworks.

## Introduction

Keeping in mind the end goal to meet the exceptional prerequisites for high caliber of administration (QoS) and high data rate correspondence and in addition rising sight and sound administrations, telecom experts are as of now living up to expectations towards the fourth era (4G) remote correspondence frameworks. The orthogonal frequency division multiplexing (OFDM–MIMO), a standout amongst the most encouraging innovations, has raised a lot of consideration in perspective of the quick advancement of computerized signal handling systems and circuits as of late (Hanzo et al. 2011; Nee and Prasad 2000; Xiong 2006).

At the early advancement stage (Chang 1966, 1970; Saltzberg 1967; Chang and Gibby 1968), customary strategies as that utilized as a part of single-transporter regulation were connected to actualize OFDM–MIMO modem, which oblige various sinusoidal subcarrier oscillators and multipliers in the modulator and banks of correlators in the demodulator. The execution many-sided quality restricted the improvement of OFDM–MIMO until 1971, when the discrete Fourier change (DFT) was connected to this innovation (Weinstein and Ebert 1971). The DFT essentially rearranged the regulation and demodulation forms and made it down to earth to actualize the baseband OFDM–MIMO modem in a computerized way. From that point on, more utilizations of OFDM–MIMO have been explored by and by.

In 1980s, OFDM–MIMO was broadly concentrated on in such ranges as high-thickness recording, rapid modems, and advanced portable correspondences (Hirosaki 1981; Hirosaki et al. 1986; Cimini 1985; Alard and Lassalle 1987). Since 1990s, OFDM–MIMO has been utilized in wideband data transmission. Utilizations of OFDM–MIMO innovation incorporate lopsided computerized supporter line (ADSL), high-bit-rate advanced endorser line (HDSL), and rapid computerized supporter line (VDSL) in wired frameworks, and advanced sound television (DAB), computerized feature TV (DVB) in remote frameworks. Besides, it has likewise been perceived as the premise of the remote neighborhood (WLAN) principles (IEEE Standard 802.11a 1999; European Telecommunications Standards Institute 1996), among which the IEEE 802.11a standard is a standout amongst the most critical ones.

With the propelled improvement of advanced incorporated circuits (ICs), the high adaptability and low multifaceted nature of computerized usage of OFDM–MIMO modem has helped its application. Among various sorts of the computerized IC innovations, field programmable entryway exhibit (FPGA) has pulled in the most consideration as of late because of its prevalent execution and high adaptability. As a broadly useful IC, FPGA is a variety of doors that can be reconfigured by the originator as a flexible configuration stage. It is created taking into account the programmable rationale gadgets (PLDs) and the rationale cell cluster (LCA) idea. By giving a two-dimensional cluster of configurable rationale squares (CLBs) and programming the interconnection that associate the configurable assets, FPGA can actualize an extensive variety of math and rationale capacities (Xilinx Inc.^1^). Contrasted with other well known IC advances, for example, application particular incorporated circuits (ASICs) and computerized signal processors (DSPs), FPGA has the accompanying favorable circumstances (Cummings and Haruyama 1999; Xilinx Inc.^2^; Altera Inc.^3^; Altera Inc.^4^).

### Contribution

Reproducible estimations, in light of a wideband multipath channel emulator, exhibit the effect of different channel conditions on the achievable data rates. Besides, the examination considers the effect of chose receiver algorithms on both throughput and increment in FPGA resources, consequently highlighting conceivable tradeoffs between receiver performances.

### Comparison with existing implementations

Compared with a SISO system numerous functional units for synchronization, OFDM modulation, and channel coding units are recreated for each spatial stream. The adjustment of spatial streams, rather, is particular to MIMO receivers and requires around 30 % of the FPGA slices and half of the multipliers with direct MMSE detection. This is likewise the case contrasted with the other existing implementations.

The normal data rates are unmistakably past those achievable by single-antenna IEEE 802.11a WLAN systems, which transmit over the same transfer speed yet are constrained to a peak data rate of 54 Mbit/s (http://en.wikipedia.org/wiki/IEEE_802.11; Xilinx Inc., Item specification of Xilinx DS260 LogiCORE IP fast Fourier transform v7.1. http://www.xilinx.com/bolster/documentation/ip_documentation/xfft_ds260.pdf; Xilinx Inc., ISE 12.1 user guides. http://www.xilinx.com/bolster/documentation/dt_ise12-1_userguides.htm). The system execution is influenced by increasing so as to expand channel lengths and antenna connection/correlation. Obviously, the MIMO increase is diminished when the antenna spacing is not adequate.

By exploring chosen receiver algorithm, including parameter estimation for synchronization and channel estimation, the effect of more complex signal handling are more huge/significant and the channel estimate accuracy is improved.

## Background and motivation

In down to earth remote correspondence frameworks, the signal is not transmitted over perfect channels. It is constricted and particularly contorted by multipath engendering through transmission. Then again, the increasing so as to fade impacts can’t be repaid the transmitted signal force. In this way, keeping in mind the end goal to acknowledge dependable correspondence, it is extremely basic to assess the fading channel and even out the direct impacts in remote correspondence frameworks. The target of this theory is to outline and actualize an OFDM–MIMO framework with channel estimation and synchronization utilizing the FPGA innovation.

Configuration and execution of OFDM–MIMO-based remote correspondence framework has been considered for a long time. The greater part of the works has been concentrating on particular territories of the execution of OFDM–MIMO framework utilizing FPGA. In (2003), Dick reviewed the usage of an OFDM–MIMO handset at an abnormal state with spotlight on specific themes in the receiver outline, for example, the synchronization, packet recognition, channel estimation and balance. Garcia and Cumplido (2005), Vladimirova and Paul (2009) and Canet et al. (2012) are separately centered on the advancement of OFDM–MIMO modulator, transmitter and receiver. What’s more, OFDM–MIMO handsets outline for the AWGN direct has been displayed in Manavi and Shayan (2004), Sobaihi et al. (2010), Gopal et al. (2011). Be that as it may, there hasn’t been a far reaching work introducing a complete improvement of OFDM–MIMO framework with channel estimation and synchronization utilizing the FPGA innovation.

The fundamental goal to this work is to outline and execute a complete baseband OFDM–MIMO framework utilizing a top-down methodology, and exhibit the framework execution for different sorts of channel conditions.

## General design and implementation methodology

**Fig. 1:**
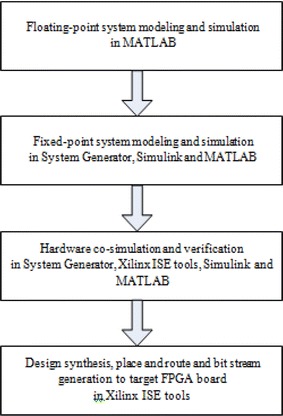
System design flow

The proposed framework is planned utilizing a top–down framework configuration approach and focused to the IEEE 802.11a standard. Framework execution will be displayed and thought about between diverse channel models. Figure 1 demonstrates the configuration stream, which incorporates four major steps.

1. Floating-point framework modeling and simulation.
2. Fix-point framework modeling and simulation.
3. Hardware co-simulation and check.
4. Design blend, place and course and bit stream era.

## OFDM–MIMO systems channel estimation design and implementation

Chapters 2 and 3 are focused on the design and implementation of basic OFDM–MIMO system and its performance under an AWGN channel. This chapter discusses the channel estimation techniques for the proposed OFDM–MIMO system. First, the modeling and classification of multipath fading are introduced. The statistics for Rayleigh and Rician channels are then discussed. In addition, typical channel estimation techniques are introduced and compared. The LS estimation algorithm is applied to the proposed OFDM–MIMO system and the BER performance over Rayleigh fading channels are simulated and compared. Finally, the receiver including estimation and equalization is implemented using FPGA.

### Wireless communication channel

**Fig. 2:**
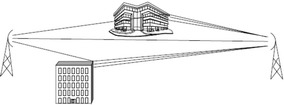
Multipath spread (Hanzo et al. 2011)

In wireless communication environments, signals may encounter reflection, refraction, and scattering during its propagation. Therefore, they arrive at the receiver through many different paths. This phenomenon is called multipath transmission as shown in Fig. 2. As a result, in spite of the ideal AWGN channel, the transmitted signals also go through the multipath fading channel in the wireless communication systems.

#### Classification of fading

The signal may experience two sorts of fading amid its spread, which are vast scale fading and little scale fading. Vast scale fading portrays the signal force misfortune over a long transmit separation. Conversely, little scale fading, which is brought about by multipath proliferation, alludes to the fast change of the signal quality over a brief time of time or separation. The fading impacts may be affected by the transmission environment, relative velocity of the receiver contrasted with that of the transmitter and encompassing items, and the relationship between the signal data transmission and the channel transfer speed too.

**Fig. 3:**
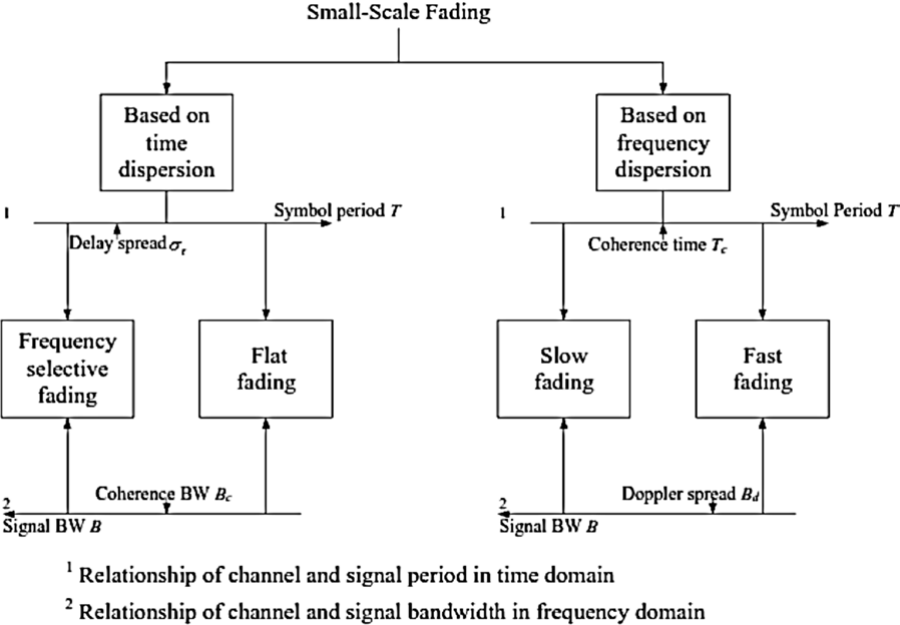
Classification of fading

In this study, we focus on the little scale fading, which is likewise called multipath fading in some writing. Regarding time scattering and frequency scattering, the little scale fading is portrayed into four sorts. The order is exhibited in Fig. 3 and will be depicted in subtle element in the accompanying.

1. Slow and fast fading

At the point when the receiver is moving amid the proliferation of the approaching flag, the range of the received signal experiences a scattering inside of a frequency extent named Doppler spread, signified by B d is characterized as (Rappaport 2002):

1 $$f_{d} = \, \frac{v}{\lambda } = \frac{v}{{c/f_{c} }} = \frac{v}{c}f_{c}$$

where v is the consistent speed of the moving receiver, λ is the wavelength of the signal, c is the pace of light, and fc is the signal bearer frequency. The shifting time nature of a channel is portrayed by Bd or intelligibility time Tc, which are characterized as

2 $$B_{d} = 2f_{d}$$

3 $$T_{c} = \, \frac{0.423}{{f_{d} }}$$

The two portray the time variety rate of the channel contrasted with signal variety. Inside of the rationality time characterized in (3), the channel is considered to have no variety.

At the point when Tc is expansive contrasted with the signal time of the transmitted signal T, the signal changes much speedier than the channel. The channel is considered as a moderate fading channel. For this situation, Doppler impacts are little or verging on insignificant. Actually, if Tc is littler than T, or the channel has a high Doppler spread, the channel fluctuates inside of a signal period, and then it is considered as a quick fading channel. To compress, the channel is moderate fading if

$$T_{c} > > T{\text{ or }}B_{d} < < \, B,$$

and it experiences fast fading when

$$T_{c} < \, T{\text{ or }}B_{d} > \, B.$$

where B = 1/T is the data transfer capacity of the transmitted signal.

2. Flat and frequency selective fading

To describe the time scattering property of a multipath fading, a couple of parameters which are conversely corresponding to one another are utilized. They are the deferral spread στ and the cognizance transmission capacity Bc. On the off chance that στ is far not as much as T, which implies the channel transfer speed is much bigger than that of the signal, the channel is known as a level fading channel. What’s more, it can be give or take displayed as a solitary Dirac capacity with a steady adequacy and straight stage reaction. Interestingly, when στ is bigger than T, the channel will have frequency particular fading impacts on the transmitted signals. In synopsis, the channel is level fading if

$$\sigma_{\tau } \ll T\;{\text{or }}B_{c} \gg \, B,$$

experiences and it frequency specific fading when

$$\sigma_{\tau } > T{\text{ or }}B_{c} < \, B.$$

By utilizing the OFDM–MIMO strategy, the signal transfer speed is isolated into a few cuts. Regardless of the fact that the channel is frequency specific inside of the whole band, it safeguards the level fading attributes for each subcarrier cut. This viably enhances the framework vigor against time scattering in frequency particular fading channel.

#### Modeling of multipath fading channel

In the fading channel, every way has an individual postponement and constriction that are shifting with time and in this way, produces different duplicates of the transmitted signal. All these weighted deferred duplicates are included at the receiver. In this way, the baseband identical channel can be demonstrated as Tse and Viswanath (2005)

4 $$h(t,\tau ) = \sum\limits_{i} {\widetilde{{\alpha_{i} }}(t)\delta \left( {\tau - \tau_{i} (t)} \right)}$$

where δ (·) is the Dirac delta function, and αi (t) are the complex-valued attenuation and excess delay of the i-th path component at instant time t, respectively.

The excess delay is the time difference between the i-th arriving component and the first arriving one.

If the transmitted signal s(t) is sampled at sampling period T s limited to a two-sided bandwidth Bs = 1/Ts, that is, it is band-, it can be written in the discrete-time form as (Iskander 2008)

5 $$s(t - \tau ) = \sum\limits_{l} {s\left( {t - lT_{s} } \right)\sin c\left( {B_{s} \left( {\tau - lT_{s} } \right)} \right)}$$

At that point the received signal at time t is

6 $$\begin{aligned} r(t) & = h(t,\tau )*s(t) \\ & = \int\limits_{ - \infty }^{\infty } {h(t,\tau )s(t - \tau )d\tau } \\ & = \int\limits_{ - \infty }^{\infty } {h(t,\tau )\left( {\sum\limits_{l} {s\left( {t - lT_{s} } \right)\sin c\left( {B_{s} \left( {\tau - lT_{s} } \right)} \right)} } \right)d\tau } \\ & = \sum\limits_{l} {s\left( {t - lT_{s} } \right)} \int\limits_{ - \infty }^{\infty } {h(t,\tau )\sin c\left( {B_{s} \left( {\tau - lT_{s} } \right)} \right)d\tau } \\ \end{aligned}$$

where * signify convolution operation. Substituting (4) into (6) and in the wake of streamlining, we get

7 $$r(t) = \sum\limits_{l} {s\left( {t - lT_{s} } \right)} \sum\limits_{i} {\widetilde{{\alpha_{i} }}(t)\sin c\left( {B_{s} \left( {\tau_{i} (t) - lT_{s} } \right)} \right)}$$

Characterizing the tap-pick up hl(t) as:

8 $$h_{l} (t) = \sum\limits_{i} {\widetilde{{\upalpha_{i} }}(t)\sin c\left( {B_{s} \left( {\uptau_{i} (t) - lT_{s} } \right)} \right)}$$

we have

9 $$r(t) = \sum\limits_{l} {h_{l} (t)s\left( {t - lT_{s} } \right)}$$

**Fig. 4:**
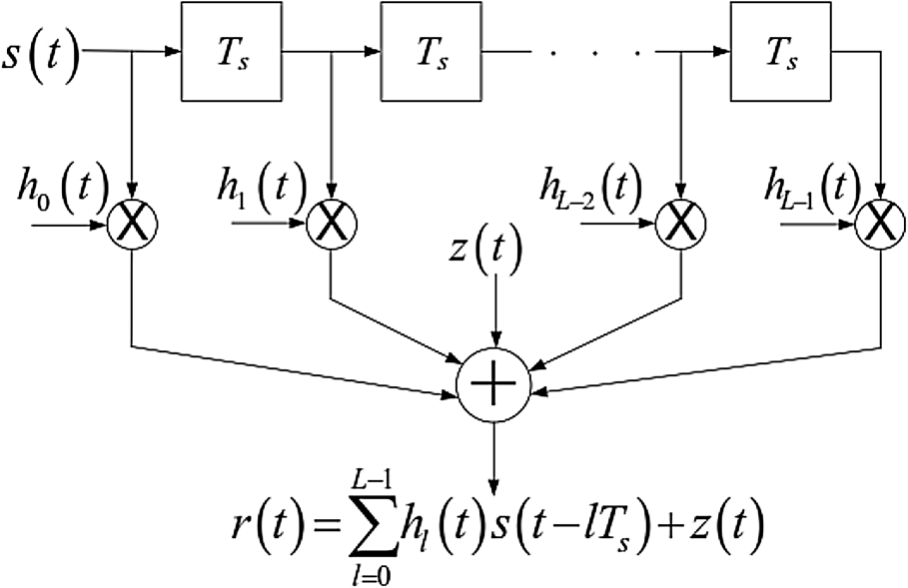
Modeling of the multipath channel utilizing similarly separated TDL

Subsequently, the channel can be displayed as a tapped-deferral line (TDL) with equivalent dividing Ts. A down to earth TDL model with length L is indicated in Fig. 4, where z(t) is the added substance clamor.

Inspecting at the same example time of the transmitted signal Ts representation of the received signal is given by, the discrete-time

In the exceptional situation when the way lessening brief time of time, that is, and deferrals are steady over a tap addition is rearranged to be

#### Statistics of fading channel

10 $$r(n) = \sum\limits_{l = 0}^{L - 1} {h_{l} (n)s(n - l) + z(n)}$$

In the special case when the path attenuations short period of time, that is, and delays are constant over a tap gain is simplified to be

11 $$h_{l} = \sum\limits_{i} {\widetilde{{\alpha_{i} }}\sin c\left( {\frac{{\tau_{i} }}{{T_{s} }} - l} \right)}$$

Because of the presence of a lot of disperses and the subsequent free engendering ways, when all is said in done, each multipath channel tap is the whole of countless parts and a conceivable overwhelming segment, in particular, a light-of-sight (LOS) segment. As a rule, the channel tap coefficient h l can be displayed as Proakis (2000)

12 $$h_{l} = \underbrace {{\sqrt {\frac{K}{K + 1}} \sigma_{l} e^{{j\psi_{0} }} }}_{LOS} + \underbrace {{\sqrt {\frac{1}{K + 1}} \sum\limits_{i = 1}^{I} {\sigma_{l} \alpha_{i} e^{{j\psi_{i} }} } }}_{NLOS}$$

where Rician variable K is characterized as the force proportion of the non-observable pathway (NLOS) parts, σI is the normal plentifulness and autonomous of time, and σi indicates the standardized genuine abundance of every segment fulfilling

$$\sum\limits_{i = 1}^{I} {\upalpha_{\text{i}}^{2} } = \, 1$$

The stage for every way is

13 $$\psi_{i} = 2\pi f_{c} \tau_{i} (t) + \varphi_{i} = 2\pi f_{d} \cos (\theta_{i} )t + \varphi_{i} \quad i = 0,1, \ldots ,I$$

in which f d is the most extreme Doppler movement, θi is the point between the bearing of the receiver movement and the waveform landing, which is free and indistinguishably circulated (i.i.d.), and φi is i.i.d., and takes after uniform conveyance on(−π, π]. Thusly, the NLOS parts can be demonstrated as round symmetric complex irregular variables (Tse and Viswanath 2005) with the entirety of standardized force

$$E\left[ {\left| {h_{l} } \right|^{2} } \right] = \sigma_{l}^{2} .$$

When I → ∞, as indicated by as far as possible hypothesis, every channel tap can be portrayed as a complex-esteemed Gaussian process. While there is no prevailing segment, which implies the Gaussian procedure has a zero mean, the baseband signal envelope |hl| accommodates with the Rayleigh conveyance. On the off chance that there is an overwhelming segment, the Gaussian procedure has a non-zero mean; the envelope takes after the Rician dissemination.

1. Rician fading

At the point when there is a stationary predominant segment, for example, a LOS signal, various arbitrary variables brought on by multipath engendering are superimposed on this prevailing signal. The channel can be displayed as a non-zero mean complex Gaussian process. Its envelope takes after the Rician conveyance with the accompanying likelihood thickness capacity (pdf),

14 $$p\left( z \right) = \, \left\{ \begin{array}{ll} \frac{z}{{\sigma^{2} }}\exp \left[ { - \frac{{z^{2} + \alpha_{0}^{2} }}{{2\sigma^{2} }}} \right]I_{0} \left( {\frac{{\alpha_{0} z}}{{\sigma^{2} }}} \right),& \quad for \, z \, \ge \, 0,\;\alpha_{0} \ge 0 \hfill \\ 0,& \quad otherwise \hfill \\ \end{array} \right.$$

**Fig. 5:**
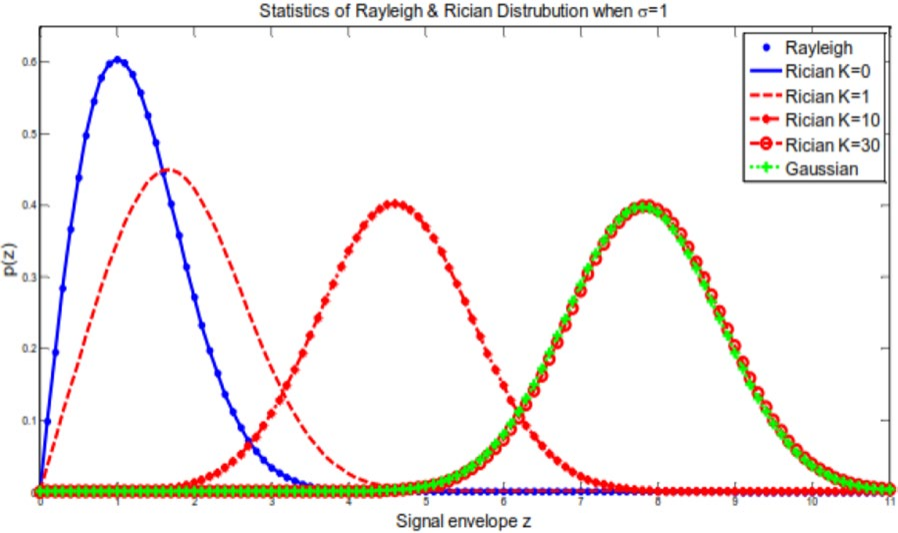
The pdfs of Rayleigh and Rician circulations

From Fig. 5, it is evident that when K → 0 force of the LOS way diminishes and can be considered as a NLOS way, the Rician circulation transforms into Rayleigh conveyance; when K → ∞, the force of the LOS way increments and the Rician appropriation transforms into a Gaussian dispersion. In the proposed outline, a poor channel environment is expected without a LOS way. In this way, Rayleigh dispersion is connected.

2. Rayleigh fading distribution

Rayleigh circulation is typically utilized for depicting the measurements of the envelope for level fading signal, or that of an individual multipath channel tap (Rappaport 2002). It has a pdf portrayed by

15 $$p\left( z \right) \, = \, \left\{ \begin{array}{ll} \frac{z}{{\sigma^{2} }}\exp \left[ { - \frac{{z^{2} }}{{2\sigma^{2} }}} \right],&\quad for \, z \, \ge 0 \hfill \\ 0,&\quad otherwise \hfill \\ \end{array} \right. \,$$

The squared extent γ = z2 is exponentially rotted with a pdf of

16 $$p(\gamma ) = \frac{1}{{2\sigma^{2} }}e^{{ - \frac{\gamma }{{2\sigma^{2} }}}} ,\quad \gamma \ge 0$$

### Channel estimation and equalization for OFDM–MIMO system

As talked about some time recently, the transmitted signal will encounter debasement as far as sufficiency constriction and stage variety amid its proliferation along a multipath fading channel. These weaknesses will bring about a huge corruption in the framework execution contrasted with that in an AWGN channel. Generally, intelligible demodulation is embraced at the receiver, as it accomplishes an execution superior to anything that of a non-lucid demodulation plan as far as BER.

For reasonable demodulation, it is obliged that we have the information of channel varieties so that the channel impact could be repaid at the receiver. This procedure is called channel estimation and evening out (Xiong 2006).

#### Channel estimation methods for OFDM–MIMO system

Fundamentally, channel estimation routines can be grouped into three classes: pilot-helped (PA), choice coordinated (DD), and visually impaired.

In the visually impaired channel estimation approaches, a lot of got information is obliged to dissect the insights of the received signal that are used to gauge the channel. In spite of the fact that the unlucky deficiency of pilot enhances the data transmission proficiency, the use of visually impaired estimation is restricted to time-shifting channels (Han et al. 2003) for it obliges complex calculation and experiences moderate union. Contrasted with PA channel estimation techniques, blind estimation systems more often than not have more terrible execution, particularly in quick fading channels.

The PA and DD channel estimation may apply the same estimation calculations.

The distinction is the wellspring of the data to the estimator (Xiong 2006). In the PA channel estimation, the pilot signals named introductions or pilot subcarriers are transmitted occasionally crosswise over time or frequency pivot. With the learning of the pilots, the receiver can extricate the channel data for the committed time and subcarriers. In the wake of applying different interjection strategies, the channel gauges for particular time and subcarrier can be acquired.

DD channel estimation, notwithstanding, uses the channel gauges for past signals to demodulate the current OFDM–MIMO signal. In such systems, all the subcarriers of each OFDM–MIMO signal are utilized to evaluate the channel. Since the pilots are not utilized, the transfer speed and force misfortune acquired by pilots PA strategies are diminished. On the other hand, the amassing of estimation slips makes it not as dependable as the PA techniques. In the event that the channel changes essentially inside adjoining signals, the framework execution may debase extraordinarily.

Indeed, even in the DD channel estimation strategies, a few preludes or pilots are obliged to perform a beginning estimation. The PA estimation methods are generally embraced in many remote interchanges, particularly the burst correspondence framework, since it can accomplish solid estimation precision in a brief while. In the framework outline, the tradeoff among estimation exactness, pilot length and thickness, and signal throughput and force is normally considered.

#### Pilot-helped estimation for OFDM–MIMO system

**Fig. 6:**
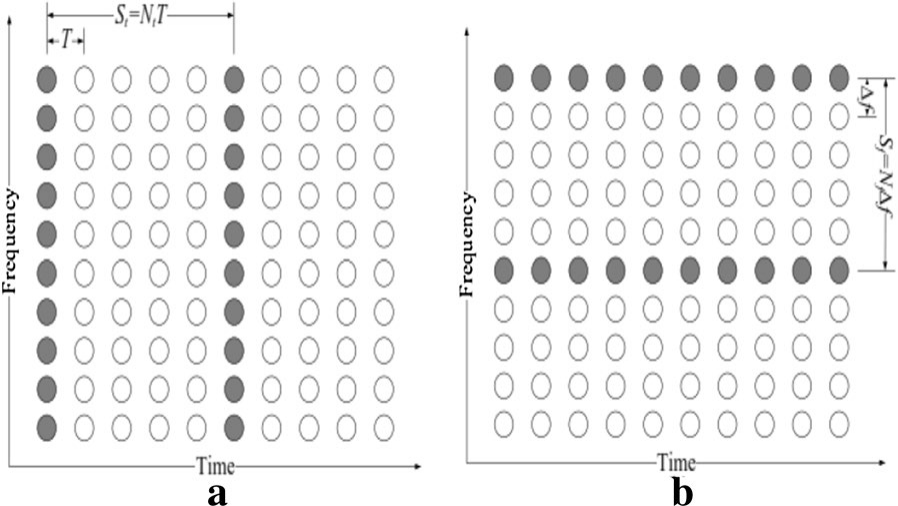
Pilot course of action for **a** square sort and **b** brush sort (Cho et al. 2010)

In PA estimation systems, pilots are embedded in time and frequency spaces in specific examples. The two essential sorts of 1D examples introduced in Fig. 6 are square sort and brush sort. They can be consolidated to shape distinctive 2D examples as demonstrated in (Tufvesson and Maseng 1997).

For square sort, the pilots are embedded into all the subcarriers in each span of time to shape an exceptional class of signals named prefaces or preparing signals, checked as strong circles in Fig. 6a. This sort is suitable for the estimation of a frequency specific fading channel, however not proficient for a quick fading channel. To enhance the framework execution over a quick fading channel, certain subcarriers in every signal ought to be held as pilots. This is the brush sort demonstrated in Fig. 6b.

To evaluate the time changing qualities, the channel ought to stay invariant inside of the cognizance time

$$T_{c} \approx 1/B_{d} .$$

At the end of the day, the pilot period should be littler than Tc. Characterize St to be the pilot separating in time (Xiong 2006):

17 $$S_{t} = N_{t} T$$

where Nt is the dispersing between nearby pilot signals in time area, and T is the OFDM–MIMO signal time.

For fruitful estimation, it ought to fulfill (Nee and Prasad 2000)

18 $$S_{t} < \, \frac{1}{{B_{d} }}$$

Thus, with the end goal of portraying channel frequency variety, the pilot dispersing Sf in the frequency space must be littler than the rationality transfer speed, which is conversely extent to the channel deferral spread σ_τ_.

19 $$S_{f} < \, \frac{1}{{\sigma_{\tau } }}$$

where

20 $$S_{f} = N_{f} \Delta f$$

The LS estimation is the most crucial technique in pilot-helped calculations (Yuan et al. 2008). As the proposed examination is taking into account the burst correspondence framework depicted in 802.11a, the channel is expected to continue as before over the season of a burst (Dick and Harris 2003). For this situation, LS estimation is fitting to be connected. Albeit different calculations, for example, least mean-square-slip (MMSE) perform superior to anything LS, it is embraced in the proposed framework for its low usage intricacy.

Assume S is the transmitted signal in the frequency area, R is the frequency reaction of the received signal, H is the frequency motivation reaction of the fading channel, H is the evaluation of H, and Z is the added substance commotion. At that point,

21 $${\mathbf{R}} = {\mathbf{H}} \cdot {\mathbf{S}} + {\mathbf{Z}}$$

in which

$$\begin{aligned} {\mathbf{R}} &= \left[ {R_{0} ,R_{1} , \ldots ,R_{N - 1} } \right]^{T} \hfill \\ {\mathbf{S}} &= diag\left[ {S_{0} ,S_{1} , \ldots , S_{N - 1} } \right],\quad {\text{with}}\;E\left\{ {S_{k} } \right\} = 0\;{\text{and}}\;Var\left\{ {S_{k} } \right\} = \sigma_{s}^{2} \hfill \\ {\mathbf{H}} &= \left[ {H_{0} ,H_{1} , \ldots ,H_{N - 1} } \right]^{T} \hfill \\ {\mathbf{Z}} &= \left[ {Z_{0} ,Z_{1} , \ldots , Z_{N - 1} } \right]^{T} ,\quad {\text{with}}\;E\left\{ {Z_{k} } \right\} = 0\;\;{\text{and}}\;Var\left\{ {Z_{k} } \right\} = \sigma_{z}^{2} \hfill \\ \end{aligned}$$

where diag [·] signifies the askew lattice and [·]T are the mean and change, individually. The essential thought of LS estimation is to discover the assessments of the channel that minimizes the expense capacity

$$J\left( {\widehat{{\mathbf{H}}}} \right)$$

as given by

22 $$J\left( {\widehat{{\mathbf{H}}}} \right) \, = \left\| {{\mathbf{R}} - \widehat{{\mathbf{H}}}{\mathbf{S}}} \right\|^{2} = \, \left( {{\mathbf{R}} - \widehat{{\mathbf{H}}}{\mathbf{S}}} \right)^{H} \left( {{\mathbf{R}} - \widehat{{\mathbf{H}}}{\mathbf{S}}} \right)$$

where [·]^T^ means conjugate transpose. By compelling

$$\frac{{\partial J\left( {\widehat{{\mathbf{H}}}} \right)}}{{\partial \left( {\widehat{{\mathbf{H}}}} \right)}} = 0$$

We get the fancied

23 $$\widehat{{\mathbf{H}}} = {\mathbf{S}}^{ - 1} {\mathbf{R}}$$

The mean square slip (MSE) is a critical parameter that portrays the execution of a calculation. It is characterized by

24 $$MSE = E\left\{ {\left( {{\mathbf{H}} - \widehat{{\mathbf{H}}}} \right)^{H} \left( {{\mathbf{H}} - \widehat{{\mathbf{H}}}} \right)} \right\}$$

By substituting (23) in (24), we get

25 $$MSE = \frac{{E\left\{ {{\mathbf{Z}}^{H} {\mathbf{Z}}} \right\}}}{{E\left\{ {{\mathbf{S}}^{H} {\bf S}} \right\}}} = \frac{{\sigma_{z}^{2} }}{{\sigma_{s}^{2} }}$$

### MATLAB simulation results for an OFDM–MIMO system

#### Simulation of IEEE 802.11a channel

As indicated by examination in “Wireless communication channel” section, the multipath fading channel is demonstrated as a limited motivation reaction (FIR) channel. For an indoor remote channel, the channel tap increases adjust to Rayleigh appropriation. The normal force deferral profile takes after exponential model (Nee and Prasad 2000)

26 $$p_{l} = p_{0} \cdot e^{{ - \beta_{0} l}} ,\quad l = 0, \ldots ,L - 1$$

what’s more, fulfills

$$\sum\limits_{l = 0}^{L - 1} {p_{l} = 1}$$

where p0 is the normal force of first tap, β0 is a parameter dictated by the examining Ts and the deferral spread στ, and L is the channel length.

The channel is modeled as

27 $$h\left( t \right) \, = \sum\limits_{l = 0}^{L - 1} {\sqrt {p_{l} } h_{l} \delta \left( {t - lT_{s} } \right)}$$

where h l is an i.i.d. complex Gaussian irregular variable with 802.11 channel with σ2 = ½.

**Fig. 7:**
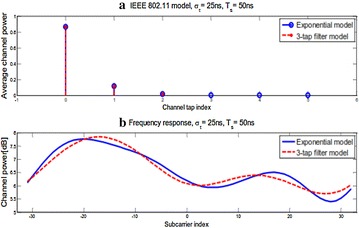
Simulation of IEEE 802.11 channel **a** normal channel force and **b** frequency reaction

A regular IEEE 802.11 channel with σ = 25 ns and Ts = 25 ns is demonstrated in Fig. 7.

The channel is thought to be invariant in one OFDM–MIMO outline which comprises of 10 OFDM–MIMO signals. The channel tap increases changes for each OFDM–MIMO outline. The edge size is set to be generally little to check the framework execution in a serious correspondence environment. In pragmatic indoor interchanges, it is appropriately decided to lessen the overhead. For usage straightforwardness, the initial three taps are utilized to produce the proposed channel. From the frequency reaction demonstrated in Fig. 7b, we can watch that the 3-tap FIR channel model keeps the sweeping statement of the wanted channel.

#### LS estimation for OFDM–MIMO system based on 802.11a

For the indoor correspondence framework in light of IEEE 802.11a, the channel is thought to be invariant in one casing whose period goes on for a few OFDM–MIMO signals. In this manner, the LS strategy in light of square sort orchestrated pilots is connected. The channel is evaluated from the long preparing succession represented in Fig. 6. It incorporates two indistinguishable LTSs.

Every signal has 64 specimens in the time area and comprises of 53 subcarriers (counting dc) as showed beneath.

As

$$L_{ - 26,26} = \left\{ {\begin{array}{*{20}l} { + 1, + 1, - 1, - 1, + 1, + 1, - 1, + 1, - 1, + 1, + 1, + 1, + 1, + 1, + 1, - 1, - 1, + 1,} \\ { + 1, - 1, + 1, - 1, + 1, + 1, + 1, + 1,0, + 1, - 1, - 1, + 1, + 1, - 1, + 1, - 1, + 1,} \\ { - 1, - 1, - 1, - 1, - 1, + 1, + 1, - 1, - 1, + 1, - 1, + 1, - 1, + 1, + 1, + 1, + 1} \\ \end{array} } \right\}$$

**Fig. 8:**
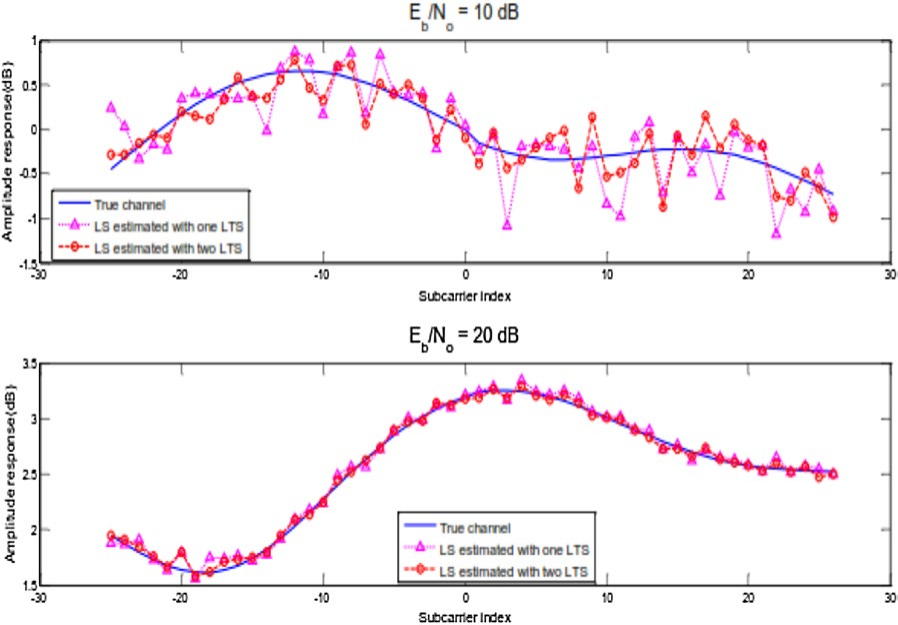
Comparison of channel reaction in the middle of assessments and genuine channel

The evaluations of the channel frequency reaction is reworked by (23)

28 $$\widehat{{\mathbf{H}}} = {\mathbf{L}}_{{\mathbf{X}}}^{ - 1} {\mathbf{L}}_{{\mathbf{Y}}}$$

where LX and LY are the transmitted and got long signals in the frequency space, individually. To further enhance the framework execution, the normal of the two got LTSs is utilized to ascertain the channel gauges.

29 $$\widehat{{\mathbf{H}}} = \frac{1}{2}\left( {{\mathbf{L}}_{{\mathbf{X}}}^{ - 1} {\mathbf{L}}_{{{\mathbf{Y1}}}} + {\mathbf{L}}_{{\mathbf{X}}}^{ - 1} {\mathbf{L}}_{{{\mathbf{Y2}}}} } \right) = \frac{1}{2}{\mathbf{L}}_{{\mathbf{X}}}^{ - 1} \left( {{\mathbf{L}}_{{{\mathbf{Y1}}}} {\mathbf{ + L}}_{{{\mathbf{Y2}}}} } \right) = {\mathbf{H}} + \frac{1}{2}\left( {{\mathbf{Z}}_{1} + {\mathbf{Z}}_{2} } \right)$$

in which LY1 and L individually, and ZY21 are the frequency changes of the first and second got LTS, and Z2 are the added substance commotion of the two LTSs. Figure 8 thinks about the frequency reaction between the genuine channel and the appraisals at distinctive clamor values. The examination is performed along all the 52 non-invalid subcarriers. From this figure, we can infer that the deviation between the two reactions is diminished with expanding Eb/N0. Regardless, the estimation with two LTSs yields preferred results over that with one LTS. Be that as it may, the change is decreased at high Eb/N0.

Moreover, when two LTSs are utilized, MSE is lessened to one-a large portion of that utilizing stand out LTS. As per (25),

30 $$\left\{ \begin{aligned} MSE_{1LTS} &= \frac{{\sigma_{z}^{2} }}{{\sigma_{s}^{2} }},\quad when \, one \, LTS \, is \, used \hfill \\ MSE_{2LTS} &= \frac{{\sigma_{z}^{2} }}{{2\sigma_{s}^{2} }},\quad when \, two \, LTS \, are \, used \hfill \\ \end{aligned} \right.$$

**Fig. 9:**
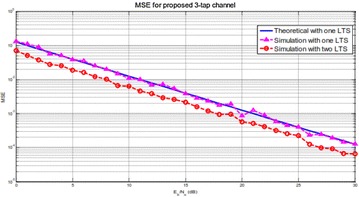
MSE correlation

From Fig. 9, it is seen that the reproduced MSE matches the hypothetical one given by (30). At the point when two LTSs are utilized to gauge the channel, MSE execution enhances by around 3 dB contrasted with that utilizing stand out LTS, which is the same as given by the hypothetical examination.

#### Simulation of BER performance

As per the investigation given in “LS estimation for OFDM–MIMO system based on 802.11a” section, the BER of the OFDM–MIMO framework is the same as that for a solitary transporter framework without ISI and ICI. At the point when the lucid QPSK adjustment is performed, the quick BER is considered as restrictive lapse likelihood given that the received signal to clamor proportion (SNR) γ_b_ is known (Proakis 2000).

As per (2.12), it is communicated as

31 $$P\left( {E|\gamma_{b} } \right) = Q\left( {\sqrt {2\gamma_{b} } } \right)$$

where γ_b_ is additionally an irregular variable as we can regard the fading channel as an AWGN channel with an arbitrary increase hl and given by

32 $$\gamma_{b} = \, \frac{{\left| {h_{l} } \right|^{2} E_{b} }}{{N_{0} }}$$

For BER averaging, fade channel is received

$$P\left( {E|\gamma_{b} } \right)$$

over the pdf of γ_b_

33 $$P_{b} = \int\limits_{0}^{\infty } {P\left( {E|\gamma_{b} } \right)p(\gamma_{b} )d\gamma_{b} }$$

At the point when the channel is level and gradually fading, the fading coefficient h1 takes after the Rayleigh appropriation with standard deviation 2σ2. Likewise, h12 has a Chi-square circulation with two degrees of flexibility with pdf indicated in (16). Along these lines, γ_b_ is likewise Chi-square appropriated. Characterize

34 $$\overline{\gamma }_{b} = E(\gamma_{b} ) = E\left( {h_{1}^{2} } \right)\frac{{E_{b} }}{{N_{0} }} = 2\sigma^{2} \frac{{E_{b} }}{{N_{0} }}$$

At that point, the pdf of γ_b_ can be composed as

35 $$p(\gamma_{b} ) = \frac{1}{{\overline{\gamma }_{b} }}e^{{ - \frac{{\gamma_{b} }}{{\overline{\gamma }_{b} }}}}$$

By substituting (35) into (33) and performing the coordination, we get

36 $$P_{b} = \frac{1}{2}\left( {1 - \sqrt {\frac{{\overline{\gamma }_{b} }}{{\overline{\gamma }_{b} + 1}}} } \right)$$

**Fig. 10:**
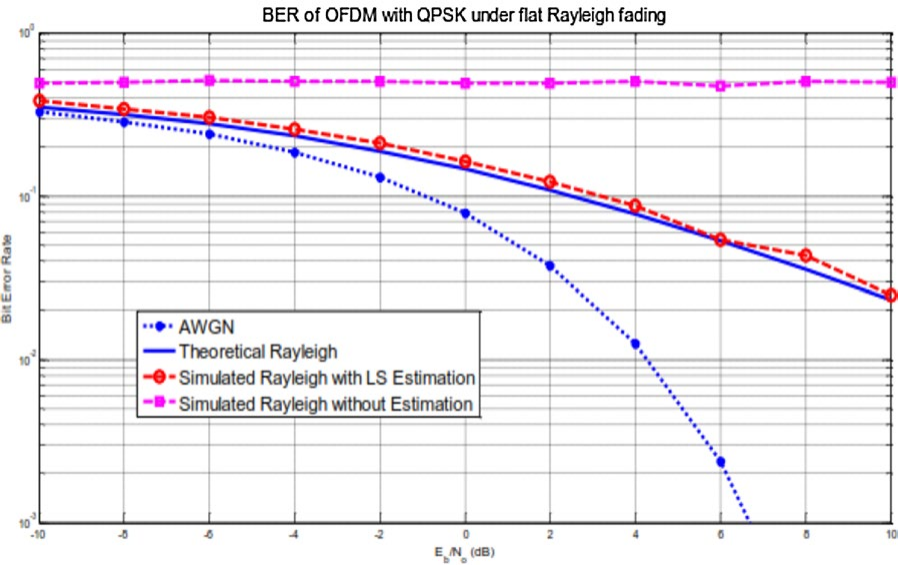
BER performance of an OFDM–MIMO system under Rayleigh fading channel

The BER execution of a Rayleigh fading channel is contrasted with that of an AWGN divert in Fig. 10. It is watched that for a fading channel, if the channel estimation is not performed, the BER is right around 0.5, which is high, and can’t get enhanced when Eb/N0 is expanded. A BER examination of AWGN and Rayleigh fading channels states that the fading impacts debase the framework execution particularly at high Eb/N0. Likewise, when the LS estimation is connected, the BER execution got a critical change, and the change increments as Eb/N0 goes higher. Then again, the simulation results demonstrate a corruption of around 0.5 dB contrasted with the hypothetical qualities.

To illustrate the BER performance over a frequency selective channel, Table 1 lists five kinds of fading channels with various models. For implementation simplicity, the frequency selective channel is modeled as a 3-tap FIR filter. Each channel tap conforms to the Rayleigh distribution. According to “Simulation of IEEE 802.11a channel” section, the underlying IEEE 802.11 channel is the fourth one in Table 1.

**Table 1:** Five kinds of fading channels

Channel index	Average power model	Average power proportion	Total of received power
1	Flat	1	1
2	Equal	1:1:1	1
3	Halfly decayed	1:0.5:0.25	1
4	Exponentially decayed	$1{:}e^{{ - \beta_{0} }}{:}e^{{ - 2\beta_{0} }}$	1
5	Exponentially decayed	$1{:}e^{{ - \beta_{0} }}{:}e^{{ - 2\beta_{0} }}$	2

**Fig. 11:**
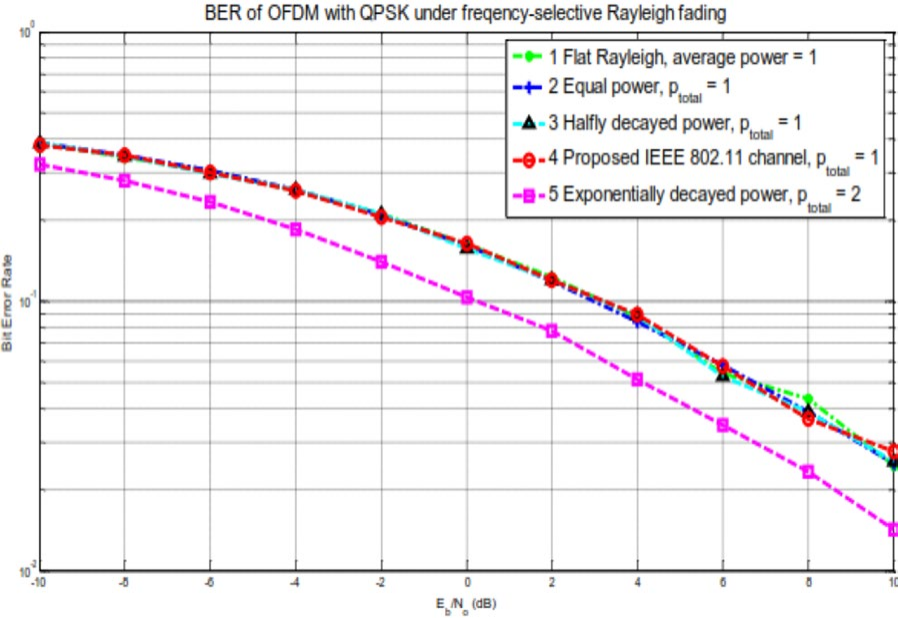
BER execution of an OFDM–MIMO framework under frequency particular channels

The simulation results plotted in Fig. 11 show that the BER performance remains almost the same for the channels with the same total received power, no matter what kind of channel model is applied. When the total power is doubled, the BER is enhanced by nearly 3 dB.

### FPGA implementation of a LS estimator

#### Modeling of the receiver with LS estimation

The channel frequency reaction is assessed utilizing LS calculation. The long preparing succession is utilized to perform the estimation after the synchronization. In this exploration, preferably culminate synchronization is expected.

Utilizing (28) and (29), the two indistinguishable long signals are arrived at the midpoint of to enhance the estimation quality. To diminish the computation unpredictability, the normal is performed in the time area before FFT preparing. The outcome is not influenced in light of the fact that the FFT change is direct. Utilizing L space, we have y1 and Ly2 to speak to the two got LTSs in the time area, we have

37 $${\mathbf{L}}_{{\mathbf{Y}}} = \frac{1}{2}\left( {{\mathbf{L}}_{{{\mathbf{Y1}}}} + {\mathbf{L}}_{{{\mathbf{Y2}}}} } \right) = \frac{1}{2}\left[ {FFT\left( {L_{y1} } \right) + FFT\left( {L_{y2} } \right)} \right] \, = FFT\left( {\frac{{L_{y1} + L_{y2} }}{2}} \right)$$

The channel impacts are evened out by applying zero driving (ZF) system. Review that R is the frequency change of the received OFDM–MIMO signals. The estimation of transmitted signal is gotten by isolating the received signals in frequency area by assessed channel reaction. Since the division estimation is convoluted and asset expending, it is refined by complex duplication without execution diminishment.

In this manner, we get the accompanying assessed frequency domain symbol,

38 $$\widehat{{\mathbf{S}}} = \widehat{{\mathbf{H}}}^{ - 1} {\mathbf{R}} = \frac{{{\mathbf{L}}_{{\mathbf{X}}} }}{{{\mathbf{L}}_{{\mathbf{Y}}} }}{\mathbf{R}} = \frac{{{\mathbf{L}}_{{\mathbf{X}}} }}{{\left| {{\mathbf{L}}_{{\mathbf{Y}}} } \right|^{2} }}{\mathbf{L}}_{{\mathbf{Y}}}^{*} \cdot {\mathbf{R}}$$

For QPSK demodulation utilizing hard choice system, S are contrasted and limit “0” to figure out if the transmitted information is bit 0 or 1. Along these lines, the circuit could be further improved by evading the division of LTS force.

39 $${\hat{\mathbf{S}}} = {\mathbf{L}}_{{\mathbf{X}}} \cdot {\mathbf{L}}_{{\mathbf{Y}}}^{*} \cdot {\mathbf{R}}$$

**Fig. 12:**
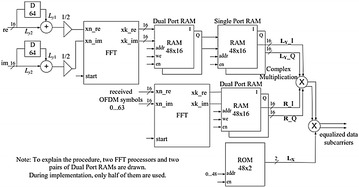
The execution of the OFDM–MIMO receiver including channel estimation and evening out

The square graph that executes the LS estimator is shown in Fig. 12. In the first place, the normal of LTS in the time space is ascertained. After FFT, L acquired. As expressed in “MATLAB simulation results for an OFDM–MIMO system” section, they are sustained to the “Subcarrier Deallocation” to get the 48 information subcarriers of LY. In the meantime, the qualities at the pilot subcarriers are likewise isolated. These information subcarriers of LY are put away in two 48 × 16 single port RAMs. The aftereffect of the mind boggling duplication of LY*·R is at last reproduced by LX, which is −1 or 1, put away in a 48 × 2 ROM.

**Fig. 13:**
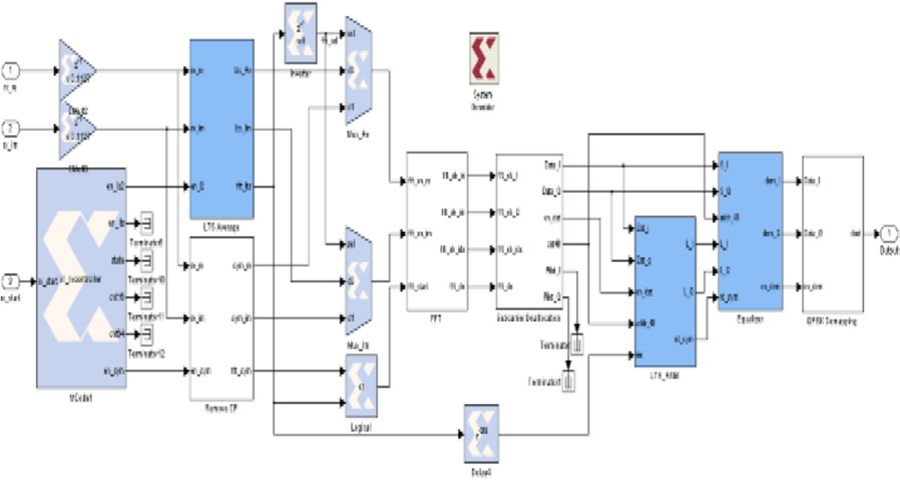
Implementation module of the channel estimation and evening out. The square, which is the equalizer, is executed as demonstrated in Fig. 14

Figure 13 demonstrates the execution of the OFDM–MIMO receiver including channel estimation and evening out. Three squares are included expansion to the essential OFDM–MIMO receiver plan in this usage graph. The “LTS_Average” square ascertains the normal of

$${\text{LTSs}}\left( {\frac{{L_{y1} + L_{y2} }}{2}} \right)$$

**Fig. 14:**
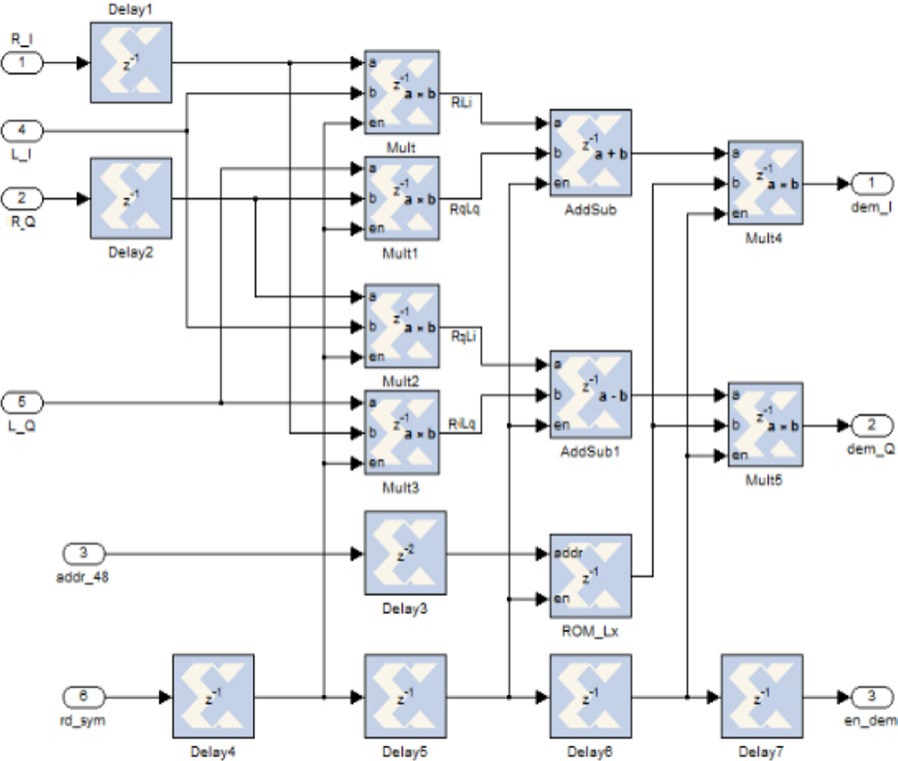
Implementation module of the equalizer

The “LTS_RAM” utilizes two single port RAMs to store the unpredictable qualities for information subcarriers of LTS (LY). The “Equalizer” performs complex augmentation of LY·LY*·R and evacuates the channel impacts for information subcarriers.

The unpredictable augmentation is executed utilizing four multipliers and two adders. The complete circuit obliges six multipliers altogether.

#### System performance

**Fig. 15:**
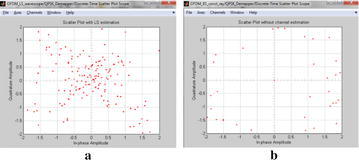
Constellation graph at the beneficiary **a** with LS estimation and **b** without channel estimation

The fading direct with file 4 in Table 1 is connected. It is displayed by 802.11 benchmarks. For execution effortlessness, the channel is based in view of a 3-tap FIR channel with every tap increase adjusting to the Rayleigh appropriation. Figure 15a, b portray the heavenly body charts at the receiver with LS estimation and without estimation, individually. The estimation of Eb/N0 is 5 dB.

**Fig. 16:**
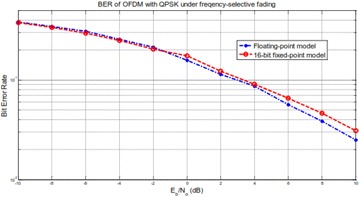
BER examination of an OFDM–MIMO framework under frequency particular fading channel

The BER versus Eb/N0 bends taking into account coasting point and settled point frameworks are shown in Fig. 16. It is watched that the BER bend of the 16-bit settled point model matches that of the coasting point model at low SNR, while the debasement happens at high SNR. The BER corruption achieves 0.9 dB at Eb/N0 = 10 dB.

#### Implementation results

Tables 2 and 3 rundown the asset utilization and timing results for the beneficiary with LS estimation, separately. It is watched that no timing clash happens in the configuration. The greatest frequency is 97.991 MHz, which is sufficient to create 40 MHz clock and drive the entire framework. By looking at Tables 1 and 2, we can infer that when the channel remuneration is executed at the receiver, the utilization of assets like cut registers, cut LUTs, fortified IOBs and Square RAMs are verging on expanded by 1 %, while the use of DSP48Es is expanded by three times that utilized as a part of the essential outline. The base period is verging on multiplied and the greatest frequency is diminished considerably. The most extreme way postpone is verging on multiplied. On the other hand, as a result of high pipelined configuration, the clock postponement stays to associate with 2 ns.

**Table 2:** Area results for receiver with LS estimation

Resources	Used	%
Number of slice registers	2806	4
Number of slice LUTs	2675	3
Number of bonded IOBs	47	7
Number of BlockRAM/FIFO	3	2
Number of BUFG/BUFGCTRLs	1	3
Number of DSP48Es	18	28

**Table 3:** Clock and timing results for receiver with LS estimation

Parameters	Time frequency
Maximum delay of clock net (ns)	2.121
Minimum period (ns)	8.287
Maximum frequency (MHz)	120.671
Maximum path delay from/to any node (ns)	8.287

## Design and FPGA implementation of OFDM–MIMO synchronization

The direct estimation system proposed in Chapter 4 is in light of the presumption that the cognizant gathering is great. That is, the framework synchronization is splendidly actualized. Synchronization is a standout amongst the most difficult and imperative errands to any beneficiary utilizing reasonable tweak, particularly for OFDM–MIMO frameworks which are exceptionally delicate to synchronization lapses. This part proposes a synchronizer based upon the deferral and connection calculation and the synchronization framework is displayed inside XSG.

Simulation results have been accommodated introducing the framework synchronization execution obviously. The execution results on FPGA are given toward the end.

### Synchronization for OFDM–MIMO

#### Introduction to OFDM–MIMO synchronization

As a rule, synchronization is partitioned into timing and frequency synchronization. Practically speaking, the oscillator does not deliver a bearer at precisely one frequency, and the transporter frequency is adjusted by irregular stage jitter (Nee and Prasad 2000). The stage clamor presented results in a confound of frequencies between the nearby oscillators at the transmitter and the receiver. Likewise, Doppler impacts in fading channel additionally causes frequency balances at the getting transporter. Subsequently, the quantities of subcarriers cycles inside of the FFT period are not whole numbers any longer, and the orthogonality property between subcarriers is not continued, bringing about ICI for an OFDM–MIMO framework. Conversely, in a solitary transporter framework, the stage commotion and frequency balances just diminish the received SNR. In this manner, OFDM–MIMO is amazingly delicate to frequency balances instead of single transporter frameworks (Xiong 2006).

Any frequency balance definitely presents ICI. This is considered as an inadequacy of the OFDM–MIMO method. Nonetheless, with the usage of frequency synchronization procedures, the loss of orthogonality could be adjusted. So the execution debasement brought on by frequency lapses could be minimized.

Instead of frequency balances, OFDM–MIMO is more inhumane to timing blunders. From the investigation given in Chapter 2, when the signal timing counterbalance is not exactly the gatekeeper interim, there is no ISI or ICI presented. In spite of the fact that the timing counterbalance may create a period shifting stage revolution to each subcarrier, it could be killed by method for channel estimation (Xiong 2006). Then again, if the timing balance is longer than the watchman interim, ISI would be presented and the orthogonality between subcarriers will never again be safeguarded. The assignment of timing synchronization is to discover the signal limits to forestall ISI and ICI.

Regardless of the fact that the timing mistakes are sufficiently little, time synchronization could enhance the framework power to multipath fading.

#### Classification of synchronization schemes

For OFDM–MIMO frameworks, it is constantly obliged that the time and frequency synchronization be proficient simultaneously (Xiong 2006). The most famous systems are the connection strategies, which use the relationship between the signal and it reproduction for synchronization.

There are two sorts of connection strategies. One is taking into account the cyclic augmentation, the other is in view of the preparation signals, likewise called preludes, which are known not collector ahead of time. Since the relationship strategies that utilization cyclic augmentation can just distinguish signal timing, yet fall flat in finding as to where a packet begins, it is just proper in show frameworks. For packet transmission with high information rate, the strategies in light of preparing signals are more suitable and dependable, as they find themselves able to track the casing, and synchronize the time and frequency coarsely in brief time.

For a burst OFDM–MIMO framework, the first synchronization undertaking is the estimation of the beginning purpose of a casing, which is called casing/packet location. The accompanying areas demonstrate the usage of an edge identifier for OFDM–MIMO packets.

### Schmidt and Cox synchronization scheme

#### Delay and correlation algorithm

With the use of the intermittent property of the short signals, cross-relationship of the received signal is utilized for identifying the beginning of the prelude. This is called deferral and connection calculation. The received signal is unpredictable connected with its deferred duplicate and summed over a sliding window. We can likewise consider it as the cross-relationship of two parts of the preparation grouping. The two parts of this grouping are indistinguishable to one another. So a factual measure of the signal, in particular,

40 $$C(n) = \sum\limits_{d = 0}^{W - 1} {r(n + d)r^{*} (n + d + D)}$$

is acquired, where n is the example number, r(n) is the receivedten signal in time area, and

D and W are the lengths of the deferral and sliding window, separately. For IEEE 802.11a application, D and W are decided to be whole number times the examples number in one STS (16).

Henceforth, if there is no OFDM–MIMO casing being gotten, the received flag just comprises of clamor. The relationship capacity C(n) is a zero-mean irregular variable. Then again, when the beginning of an OFDM–MIMO casing is gotten, C(n) is a cross-connection of indistinguishable short signals. It came to the most extreme worth in a brief while. By checking the worth change of C(n), the beginning of an OFDM–MIMO edge is resolved.

A few measuring systems have been presented. The most straightforward methodology is greatest connection (MC) (Xilinx Inc.^5^) plan. In this plan, the beginning of an edge is distinguished when C(n) ranges to most extreme. Since the deliberate vitality is not standardized inside of the synchronization window and differs in a wide range, it is difficult to choose a limit as the most extreme quality. So this system is not suitable for multipath fading correspondence and non-consistent envelope tweak (Kabulepa et al. 2002).

To manage the issue for MC approach, Schmidt and Cox proposed a MNC-based system in (Schmidt and Cox 1997). In this system, another sliding window is connected to ascertain the force of the deferred signal

41 $$P(n) = \sum\limits_{d = 0}^{W - 1} {r(n + d + D)r^{*} (n + d + D)} \sum\limits_{d = 0}^{W - 1} {\left| {r(n + d + D)} \right|^{2} }$$

The choice measurement called timing metric is acquired as

42 $$M(n) = \frac{{\left| {C\left( n \right)} \right|^{2} }}{{\left| {P\left( n \right)} \right|^{2} }}$$

where a standardization element of |P(n)2| is acquainted with thin the variance range.

Different techniques incorporate MMSE (Chevillat et al. 1987) and most extreme probability (ML) (Sandell et al. 1995) plans.

MMSE plan ascertains the normal forces of the received signal and it’s postponed duplicate, and contrasts the outcome and C(n). ML plan is an enhancement of MMSE thinking seriously about the SNR. These two plans are turned out to be effective in nonstop transmission frameworks, yet they expand false caution likelihood and usage unpredictability in burst OFDM–MIMO correspondence frameworks (Kabulepa et al. 2002).

#### MATLAB simulation of MNC scheme

The estimation of an edge begin is resolved to be at the specimen list n when the timing metric M(n) is augmented. M(n) is standardized and is free of indisputably the received signal force. An edge “Thr” identified with SNR is set. An edge is thought to be identified if the accompanying prerequisite is fulfilled.

43 $$M(n) > Thr$$

**Fig. 17:**
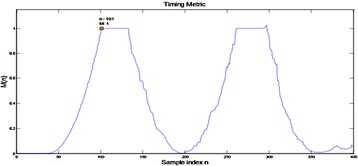
Timing metric of MNC plan in clamor free transmission

**Fig. 18:**
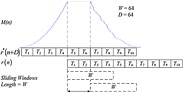
Delay and connection of short preparing grouping with W = 64

Figure 17 plots M versus test list n for the usage of MNC plan with D = W = 64 without commotion. The timing balance is 100 specimens. The general reaction is in the scope of 0–1 and the seize the edge beginning is clear. M(n) is at a low level before the beginning of an edge. When the short preparing succession is gotten, where test record n = 101, M(n) hops to the most extreme estimation of 1 rapidly. This hop gives very much a decent gauge of the casing beginning time. The level of most extreme quality closures at n = 133. The length 32 (=160 – D − W) of the level equivalents to the length of two STS periods. The outcome is examined in Fig. 18. It is demonstrated that the first example of the level concurs with the start of the short preparing arrangement. The opportune time begin is acquired. Thus, once the start of the level is identified, the time synchronization is accomplished in brief time. The second level in Fig. 17 begins at n = 261, which is the first example of the long preparing succession. Since the LTS is intermittent with period 64 and has an aggregate length of 160, there would be a comparable level of length 32.

**Fig. 19:**
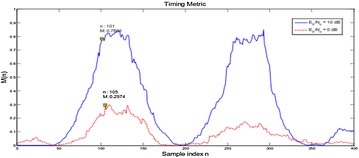
Timing metric of MNC plan under AWGN channel

Figure 19 shows M versus n bends under an AWGN channel for Eb/N0 = 10 dB and 0 dB. It is watched that the most extreme estimation of M declines with SNR. This is on account of with the increment of commotion vitality, connection between the two parts of the short preparing arrangement will diminish, yet the received force won’t. Henceforth the most extreme M and the beginning stage of casing decided as needs be differ with SNR. By contrasting the two bends, it is seen that at low SNR, the hop in the estimation of M is not as clear as that at high SNR. The side flap is moderately high, which can without much of a stretch cause false alarm.

**Fig. 20:**
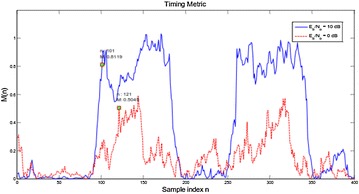
Timing metric of MNC plan under Rayleigh fading channel

Figure 20 delineates M versus n bends under a Rayleigh fading channel for Eb/N0 = 10 and 0 dB. Like the simulation aftereffects of Fig. 19, M diminishes with SNR, and the likelihood of false recognition increments altogether at low SNR. By looking at these two figures, it is watched that under multipath fading, the variety of M amid the level period is bigger than that under an AWGN channel. The recognition precision is lessened. This execution debasement is to a great degree serious at low SNR. In this manner, the MNC plan is not exactly vigorous at low SNR.

### FPGA implementation of OFDM–MIMO synchronization

#### Square diagram of OFDM–MIMO synchronization

To lessen the execution multifaceted nature, the connection capacity C(n) and P(n) could be computed with an iterative structure in a sliding window

44 $$C(n + 1) = C(n) - r^{*} (n)r(n + D) + r^{*} (n + W)r(n + W + D)$$

45 $$P(n + 1) = P(n) - \left| {r(n + D)} \right|^{2} + \left| {r(n + W + D)} \right|^{2}$$

**Fig. 21:**
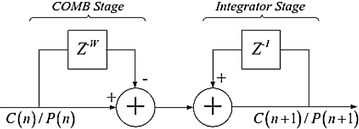
Implementation of postponement and gatherer with CIC channel

As indicated in Fig. 21, we utilize a solitary stage fell integrator-brush (CIC) channel to actualize the deferral and collection operation in (44) and (45). For instance, once the first C(n) is processed, the adding so as to accompany C(n + 1) could be executed the following cross-connection term and subtracting the first. This procedure is performed iteratively.

**Fig. 22:**
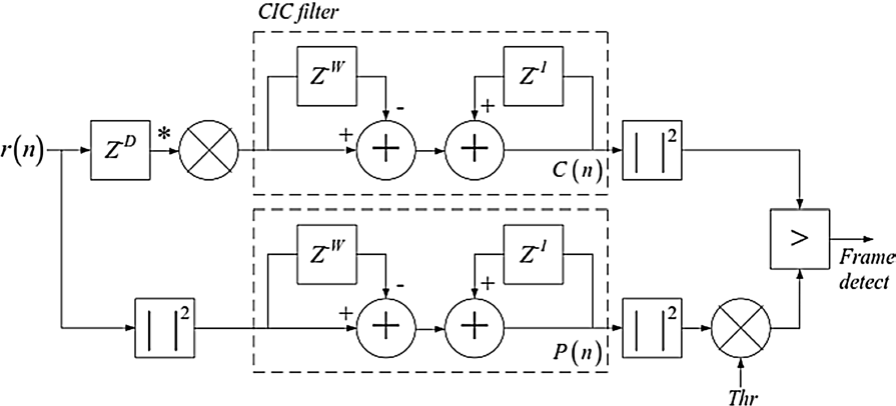
Square outline of the OFDM–MIMO synchronization

The execution of division is exceptionally asset devouring. For execution straightforwardness, we can change over the division operation to an augmentation and an edge choice. The edge recognition is accomplished when

46 $$C(n)^{2} > P(n)^{2} \cdot Thr$$

where *Thr* is figured by exploratory simulation of M(n). The decision of *Thr* is in view of SNR and the normal BER. Figure 22 demonstrates the square chart of the OFDM–MIMO synchronization, in which D = W = 64.

#### Modeling of synchronization circuit

**Fig. 23:**
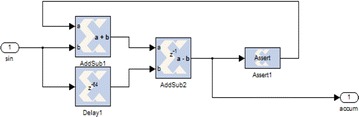
Implementation module of CIC channel

**Fig. 24:**
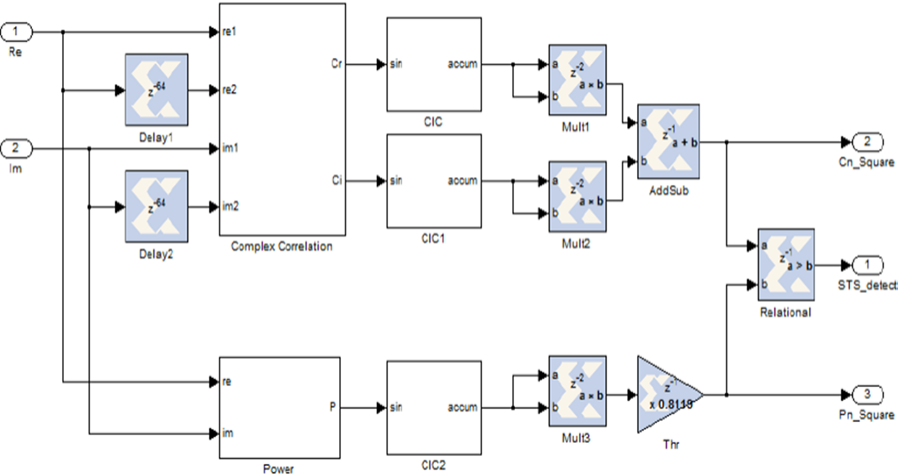
FPGA usage of the OFDM–MIMO synchronizer

From the equipment module of CIC demonstrated in Fig. 23, the collector is acknowledged by adding the new coming data to the current CIC yield. This is expert by an “Affirm” square. After amassing, a subtractor is connected to get the last result. Figure 24 shows the FPGA execution for the complete synchronizer. Three CIC channels are utilized, in which the genuine and nonexistent square of C(n) are ascertained in two branches.

**Fig. 25:**
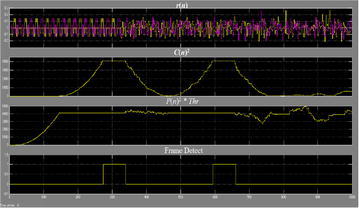
Waveforms of the synchronizer in noise-free transmission

**Fig. 26:**
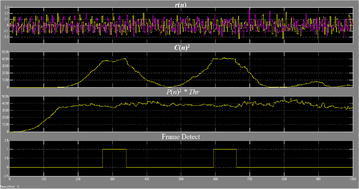
Waveforms of the synchronizer under AWGN channel at Eb/N0 = 10 dB

**Fig. 27:**
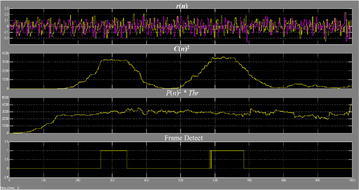
Waveforms of the synchronizer under Rayleigh channel at Eb/N0 = 10 dB

Figure 25 represents the yield waveform for a clamor free environment. It is seen that C(n) begins to develop from the fifth short signal, and accomplishes the most extreme worth amid the ninth signal. Then again, P(n) accomplishes stable state amid the fifth signal, which coordinates our hypothetical investigation. The edge is identified when test file n equivalents to 274, which is the last specimen of the eighth short signal. The principal level closures at n = 340 and goes on for a time of 33-specimen time. The second level is from n = 594 to n = 660, which is amid the last 33 specimens of the second LTS. Here every example has a time of two clock cycles. There is a length contrast of 1 specimen for the level between MATLAB simulation and FPGA usage. It is on account of for the FPGA execution, the last example of the eighth signal has a time of two clock time, while in simulation, and it is a moment point. Truth be told, for the usage waveform, the specimen distinction at the level is likewise 32, which is the same as the simulation result (Figs. 26, 27).

#### Implementation results

After the framework usefulness is checked under AWGN and Rayleigh fading channels, it is changed over to VHDL codes and afterward the union and writing computer programs are performed. The framework is modified to Virtex-5 gadget. Tables 4 and 5 compress the region and timing results. With high pipelining outline, the most extreme deferral of the clock net is additionally constrained to around 2 ns. The inactivity is 5-specimen time, which equivalents to 10 clock cycles.

**Table 4:** Area results for synchronizer

Resources	Used	%
Number of slice registers	570	1
Number of slice LUTs	793	1
Number of bonded IOBs	107	16
Number of BUFG/BUFGCTRLs	1	3
Number of DSP48Es	18	28

**Table 5:** Clock and timing results for synchronizer

Parameters	Time frequency
Maximum delay of clock net (ns)	2.064
Minimum period (ns)	7.468
Maximum frequency (MHz)	133.905
Maximum path delay from/to any node (ns)	7.468

Every specimen in time area goes on for two clock cycles. The evaluated greatest frequency is 133.905 MHz and is sufficiently expansive to drive the synchronization circuit.

Considering the asset utilization, the use of I/O and DSP48E gadget are moderately high, since three collectors are utilized.

## Conclusion

This paper has introduced the hypothetical investigation and simulation and also FPGA execution of a baseband OFDM–MIMO framework with channel estimation and timing synchronization. The OFDM–MIMO framework is prototyped in view of IEEE 802.11a standard and transmits/gets signals on a 20 MHz transmission capacity. With QPSK regulation plan, the framework accomplishes a throughput of 24 Mbps. Various types of transmitting channels have been concentrated on and different estimation and balance strategies are looked at. The customary LS calculation has been executed. For the estimation of coarse timing, a changed MNC plan has been explored and figured it out. Beginning from the investigation of OFDM–MIMO guideline, the framework has been checked and acknowledged with the assistance of both MATLAB simulation and equipment execution. The outline of the whole venture has been completed in a top-down methodology, from the framework configuration to practical squares plan. The present area gives a synopsis of the work contained in the theory.

After that, we introduce the configuration and FPGA usage of the channel estimation and adjustment subsystem for indoor remote correspondence environment.

The LS estimator is introduced and acknowledged under the proposed 802.11 channel, which is demonstrated by 3-tap FIR channel with every tap depicted by Rayleigh dissemination. By looking at the BER aftereffects of the settled point model with that of the coasting point model, it is watched that there is a BER corruption for substantial SNR because of the exactness misfortune when speaking to the frail clamor in altered bits. Then again, contrasted with that without estimation, the receiver with LS estimator enhances framework execution enormously to the detriment of expanding the asset utilization of cut registers, cut LUTs, reinforced IOBs and Square RAMs by just about 1 %. The use of DSP48Es is expanded by three times that utilized without channel estimation.

At long last, the synchronization hardware is actualized. A few connection based calculations are presented and the MNC plan which uses the cross-relationship of prelude signals is planned. Exploratory results under different correspondence situations are gotten in light of the altered point model. The execution result demonstrated the most extreme clock postponement speaks the truth 2 ns. Since the driving clock of the framework is 40 MHz, no contention exists in the planned OFDM–MIMO framework and the combined hardware meets the timing imperatives.

## Footnotes

1 http://www.xilinx.com/bolster/documentation/dt_sysgendsp_sysgen12-1.htm.

2 See footnote 1.

3 http://www.altera.com/items/programming/items/dsp/dsp-builder.html.

4 http://www.altera.com/writing/wp/wp-01023.pdf.

5 http://cdserv1.wbut.ac.in/81-312-0257-7/xilinx/documents/Xcell%20Journal%20Articles/xcell_47/xc_sysgen47.pdf.
